# Generative artificial intelligence in forensic science education: Student perspectives on AI-supported crime scene decision-making

**DOI:** 10.1016/j.fsisyn.2026.100723

**Published:** 2026-08-06

**Authors:** Paula Tarttelin Hernández, Alicia Haines, Vincent Mousseau, Madysen Elbourne, Teneil Hanna

**Affiliations:** a Centre for Forensic Science, MAPS School, University of Technology Sydney, Australia; b Department of Biochemistry, Chemistry, Physics and Forensic Science, Université du Québec à Trois-Rivières, Canada

**Keywords:** Crime scene processing and management, Professional judgement, Human-AI collaboration, AI literacy, Forensic training, Higher education, Assessment and monitoring, Teaching & learning

## Abstract

Students in Higher Education (HE) are increasingly engaging with Generative Artificial Intelligence (GenAI), raising concerns regarding learning, academic integrity, and equitable access to technology. In forensic science, these concerns are particularly significant, as professional practice relies on critical judgement, evidentiary reliability and ethical accountability. This study examined how undergraduate forensic science students engaged with a university-approved GenAI tool (Copilot) within crime scene simulations, with a focus on decision-making, ethical and responsible use, and trust. Copilot was embedded into a second-year subject (Major Scene Investigation) of a three-year undergraduate forensic science degree, as a structured decision-support tool. Students critically evaluated AI-generated outputs during simulated crime scene investigations and documented their use through reflective entries, including interaction records and reflections that could be presented in various formats (e.g. written, audio, video). These data were analysed thematically to explore student perceptions, patterns of engagement, and the development of critical AI literacy. Findings indicate that students did not adopt GenAI uncritically or as a substitute for professional judgement. Instead, they demonstrated a cautious selective approach, positioning GenAI as a conditional support tool. Engagement evolved over time, with initial scepticism giving way to more informed strategic use. Importantly, students differentiated between low- and high-stakes decisions, demonstrating reduced reliance on GenAI where accountability and evidentiary consequences were greater. Embedding GenAI within assessments supported the development of critical and ethical GenAI literacy, enabling students to articulate appropriate, cautious, and limited uses of the technology. While GenAI offered efficiencies in tasks such as hazard identification and documentation, its role remained bounded by the need for human oversight and justification. This study demonstrates the value of integrating GenAI into discipline specific learning environments to support critical engagement and understanding rather than passive use. It highlights the importance of explicitly teaching the ethical and professional boundaries of GenAI in forensic science decision-making to prepare students for responsible use of this technology in future practice.

## 1 Introduction

The integration of Generative Artificial Intelligence (GenAI) into teaching, learning, and assessment is globally revolutionising how students engage with their academic journey, with many relying on these tools to help them prepare for and complete assessment tasks [1]. GenAI technologies use deep learning models to generate novel content, including text, images, audio, and video, from input data or prompts [2,3]. These systems are effective at recognising patterns in multi-modal inputs to produce summaries, classifications, transformations, or new content that reflects the structures they have learned [2]. Their ease of use and their ability to mimic human language and respond to complex prompts makes them particularly attractive to users; however, this capability also introduces significant implications for both educational and forensic science landscapes including the risk of uncritical acceptance of AI-generated outputs, overreliance on these systems, and the delegation of thinking and decision-making to such tools [3].

Forensic science education prepares students for professional roles where critical thinking, ethical responsibility, integrity and, increasingly, digital literacy are crucial. Recent studies and reports highlight the growing application of AI across criminal justice workflows, including improvements in efficiency [4], facilitation of international collaboration, enhanced data analysis [5], and strengthened service delivery, when used appropriately as decision-support platforms under robust governance frameworks [6]. Nevertheless, stakeholders note significant concerns and ongoing uncertainty about how AI may impact privacy, autonomy, bias and discrimination, and the transparency or explainability of AI-driven decision-making [6]. Such concerns are especially salient within a justice system that demands scientific rigour and strict adherence to evidentiary standards [7]. They indicate that the primary challenge posed by AI in forensic science contexts is human-based, lying in the capacity of human decision-makers to critically evaluate, contextualise, and justify AI-assisted outputs within evidentiary and ethical frameworks [8].

The implications of AI and how it intersects with practitioner decision-making in high-stakes crime scene environments are not yet fully understood. The crime scene examination and reconstruction process is among one of the most critical stages of any investigation and is regarded as being intellectually challenging, highly demanding in nature, and decisive in guiding investigative directions [9]. In crime scene contexts, decision-making encompasses a series of interconnected decisions surrounding crime scene attendance, the search for traces, the triaging process, and the collection of traces [10,11]. Prior literature has shown that decision-making at the scene can vary broadly according to examiners’ experience, knowledge, and prior training [12] and it has been strongly recommended that improved training and guidelines are provided to assist examiners with the hypothetico-deductive mechanism for reasoning and trace utility evaluation, knowledge construction, integration, and experience acquisition [13].

Given the rapid uptake and growing interest in AI tools, particularly since the release of ChatGPT-3.5 in November 2022, examining GenAI across both educational and forensic science contexts has become increasingly necessary. Such examination is critical to ensuring that graduates are well equipped to apply these tools ethically, responsibly, and effectively, both in scholarly settings and as they transition into professional forensic practice.

As AI becomes increasingly integrated into forensic workflows, an important question arises: how do future forensic practitioners evaluate, negotiate, and integrate AI-generated information into their decision-making in complex crime scene contexts? This study presents a case example where GenAI is intentionally embedded within the assessment design of an undergraduate forensic science subject (Major Scene Investigation) that focuses on advanced crime scene examination. The aim is to support the development of students' digital and critical AI literacy and, through experiential and reflective learning, their capacity to exercise professional judgement and metacognition when engaging with AI-generated information. This includes foregrounding the ethical and responsible use of GenAI in both academic and investigative contexts, using students’ reflective insights on the integration of GenAI in higher education assessment and simulated crime scene decision-making.

### 1.1 Artificial intelligence in forensic science

The adoption of AI in forensic science is increasingly showing its transformative potential, particularly in the automation of well-defined, repetitive, and data-intensive tasks that reduce investigative burden [14]. To date, much of the literature has focused on AI to support digital forensic science, reflecting the significant big-data demands imposed by contemporary investigations [14,15]. Manual approaches to crime scene image database searching, cataloguing, and labelling have become arduous and vulnerable to subjectivity and inconsistency, particularly when operating at scale [15].

While much of the early adoption of AI has focused on digital forensic contexts, AI applications are also expanding beyond digital forensic science into other domains where human judgement has traditionally played a central role and where large databases are available, such as in fingerprint examination and forensic DNA analysis [[16], [17], [18], [19], [20]]. Under these contexts, AI offers the potential to support more consistent and scalable analytical processes, and it may reshape the conditions under which interpretative judgement is exercised, although its integration must be carefully managed to avoid reinforcing existing sources of bias.

Alongside efforts to address interpretative challenges and error, AI is also being applied more broadly across forensic science to support diagnostic and analytical decision-making. Recent reviews by Orsini et al. (2025) [21] and Garland et al. (2025) [22] highlight the expanding role of AI in forensic pathology across both diagnostic tasks and operational workflows [22]. Additional applications include the use of Convolutional Neural Networks for bruise dating [23], machine learning methods for detecting and classifying explosives [24], and illicit drug markers using sensor-based approaches [25]. Emerging technologies, such as portable near-infrared devices combined with chemometric modelling, further demonstrate the potential for real-time analysis to support and improve frontline decision-making and operational effectiveness [26]. Reports indicate that police forces are already exploring or implementing AI technologies across operational areas, including facial recognition, redaction, resource allocation, forensic intelligence, and administrative support [6].

More recently, attention has turned to the potential role of large language models (LLMs) and GPT-based architectures as decision-support tools within investigative and policing contexts. These systems have been proposed to assist with tasks such as dataset analysis, the generation of text-based responses to policing queries, and the identification of patterns across cases, although many such applications remain largely prospective [5]. LLMs have also been identified as having potential to supplement crime scene investigation workflows and predictive decision-making within the justice process [16,17].

Despite these advances, AI continues to pose significant challenges for forensic science practice. A key limitation identified across the forensic AI literature is the lack of transparency and explainability in many AI systems, which constrains their alignment with legal and regulatory standards requiring forensic decisions to be expert-led, justifiable, reviewable, and open to scrutiny [18]. Concerns relating to bias, reliability, and trust therefore continue to complicate the seamless adoption of AI within forensic science and the broader justice system. While governance frameworks are increasingly proposed to support ethical and responsible AI deployment [19], these measures are insufficient unless combined with practitioners who are capable of critically interrogating AI outputs and exercising informed, accountable judgement in their application.

### 1.2 Generative artificial intelligence in higher education

The release of ChatGPT-3.5 marked a pivotal shift in higher education, rapidly transforming the way students accessed and engaged with AI technologies. Subsequent advances in GenAI, including increasingly capable LLMs and multimodal systems, challenged traditional approaches to assessment. These developments prompted universities worldwide to reconsider assessment design and academic integrity, resulting in a series of sector-wide policy initiatives, regulatory responses, and institutional reforms.

**Fig. 1:**
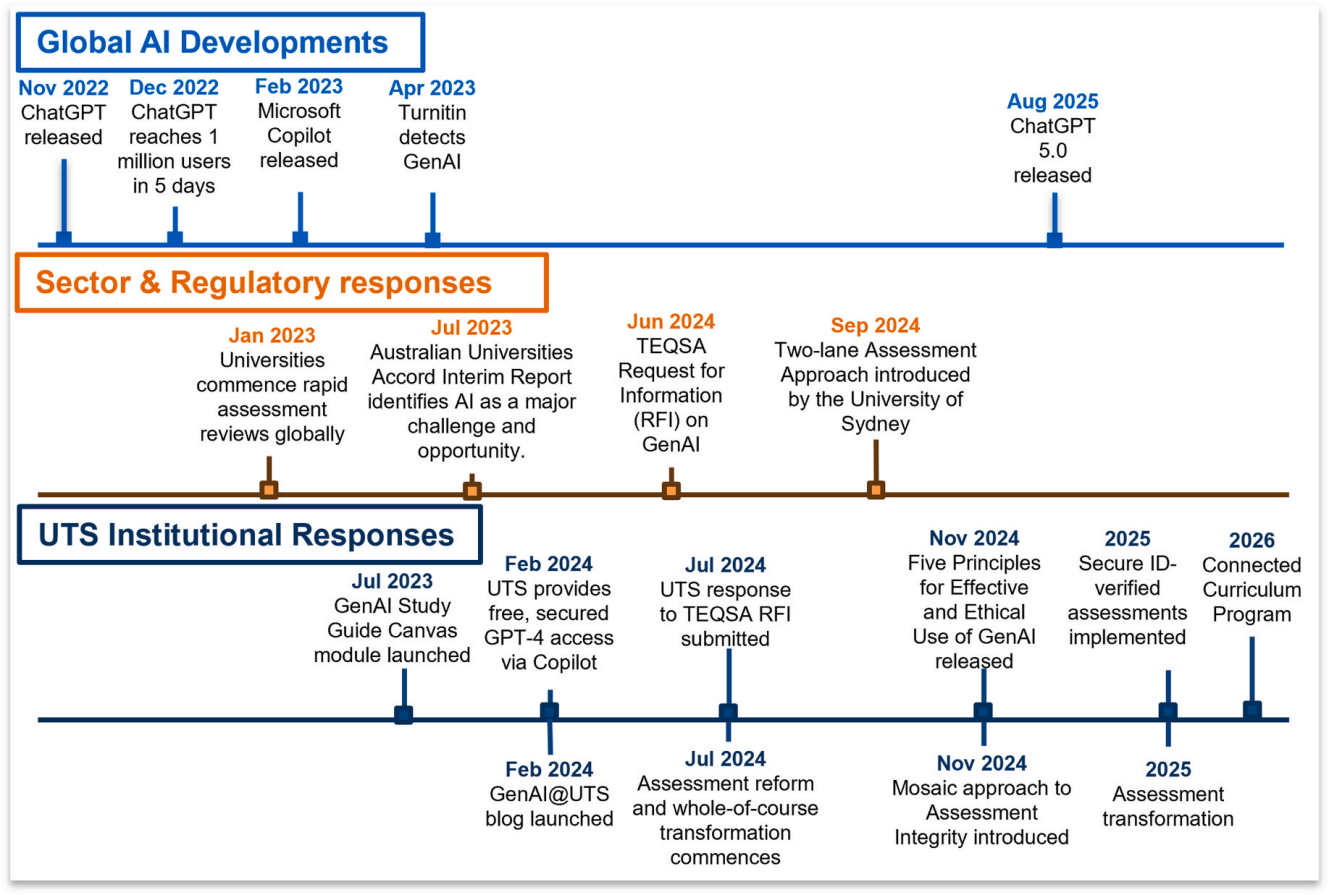
– Timeline of technological, regulatory, and institutional milestones underpinning assessment transformation at the University of Technology Sydney (see S1 for complete resource information).

Fig. 1 summarises the major technological, regulatory, and institutional milestones that shaped this evolving assessment landscape since the emergence of ChatGPT at the University of Technology Sydney (see Table S1 for resource information). The timeline provides the educational context underpinning the redesign of authentic assessments. It demonstrates how rapid advances in GenAI capability accelerated a shift from reactive approaches centred on GenAI detection (i.e. Turnitin AI detection tool) towards proactive assessment strategies that emphasise authentic learning, ethical GenAI use, professional judgement, and the development of GenAI literacy within higher education curricula.

GenAI platforms offer substantial opportunities to enhance learning in higher education through accessible, conversational interfaces capable of synthesising real-time information, generating human-like responses, and supporting students with a broad range of academic tasks. When guided by well-constructed prompts, these systems can provide instant explanations and feedback, resolve queries of varying complexity, perform onerous tasks with ease and at speed, and create new engaging content [20]. It is therefore not surprising that, as observed by Lacey and Smith (2023) [20], students are increasingly treating these systems as ‘virtual tutors’, reflecting their perceived value in supporting independent and self-directed learning. GenAI has also been identified as having potential to support inclusive and accessible learning by presenting information in various formats and at a pace and schedule that suit the individual learning needs of students [20].

Despite these benefits, widespread adoption of GenAI has introduced significant challenges for higher education. Concerns surround inappropriate reliance on these technologies and how they may affect academic integrity, circumvent intended learning outcomes, and exacerbate existing digital and educational inequities [3,27,28]. Recent surveys indicate a substantial increase in student use of GenAI, with 92 % reporting usage in 2025 (up from 66 % the year before), and 88 % using these tools specifically for assessment tasks, up from 53 % in 2024 [28,29]. Although many students report using GenAI to summarise literature, generate ideas, and explain concepts, a notable proportion (18 %) acknowledge having directly incorporated text generated by AI into submitted work [28]. These findings illustrate both the widespread adoption of GenAI and the continuing uncertainty surrounding appropriate educational use.

In response to these concerns, the Australian Tertiary Education Quality and Standards Agency (TEQSA) issued a request for information (RFI) from all registered higher education providers to propose action plans to address the risks imposed by GenAI on educational integrity. The RFI resulted in the creation of a toolkit to support institutions to implement effective strategies for ethical integration of GenAI in teaching and learning, whilst mitigating the risks to integrity posed by these tools [30], as well as a call for assessment reform in the age of GenAI [31].

Despite high adoption rates, students' digital or critical AI literacy appears to remain limited. This is compounded by students’ uncertainty about the reliability of AI outputs, limited foundational grounding, fear of being accused of academic misconduct, and confusion about institutional expectations governing acceptable and responsible AI use [28,29]. In many cases, students lack a clear understanding of how GenAI platforms function, their limitations, or how AI-generated information should be evaluated and integrated responsibly within academic and professional contexts. Addressing these gaps requires more than policy guidance alone and necessitates structured opportunities for students to engage critically with AI outputs through practical application, educator-led instruction and institutional frameworks [28,29].

The literature suggests that the central educational issue is not students' use of GenAI *per se*, but whether they are equipped to evaluate its outputs critically and exercise judgement over when, how, and to what extent AI-generated information *could* or *should* inform decision-making [30,32]. This is a challenge that becomes particularly consequential in educational forensic science and crime scene investigation contexts and it framed the motivation of this study to better understand: ‘How do undergraduate forensic science students critically engage with, evaluate, and negotiate ethical responsibility when using GenAI as a decision-support tool in simulated crime scene investigations?’.

## 2 Methodology

This study is presented and interpreted through a forensic science decision-making lens, focusing on how participants critically engaged with, evaluated, and negotiated AI-generated information within simulated crime scene investigations. It is recognised that many of the cognitive and ethical demands associated with AI-assisted decision-making emerge well before students enter professional practice and, although participants were undergraduate forensic science students, they were positioned as early-stage decision-makers operating within authentic, high-fidelity crime scene simulations designed to reflect professional investigative workflows and constraints.

A qualitative methodological approach was designed to capture students' perceptions, reasoning, and judgement formation when interacting with GenAI within crime scene decision-making contexts, using students’ reflections following the completion of the *Major Scene Investigation* subject.

### 2.1 Participant recruitment

Participant recruitment was approved by the University of Technology Sydney's ethics committee (ethics approval code: HE-2026-0238).

Part of the research team was also involved in the subject's teaching. Therefore, to mitigate the influence of power imbalances or bias, students who had completed the subject in Spring 2025 were approached to let researchers examine their reflections for research only once they had completed the subject. Participants were also reached out to via email by a research member who had not been involved with the grading of the assessments. The email contained context for the study, as well as a participant information sheet and a consent form that all participants had to read and sign before their assessments could be used for research purposes. Once this process was completed, the student reflections in Portfolium were uploaded to a secure platform and anonymised before data analysis. A total of 20 students agreed to allow the research team to evaluate their assessed reflections to better understand how they felt about the new assessment design and about using Copilot as a tool to support crime scene processes and decision-making.

**Fig. 2:**
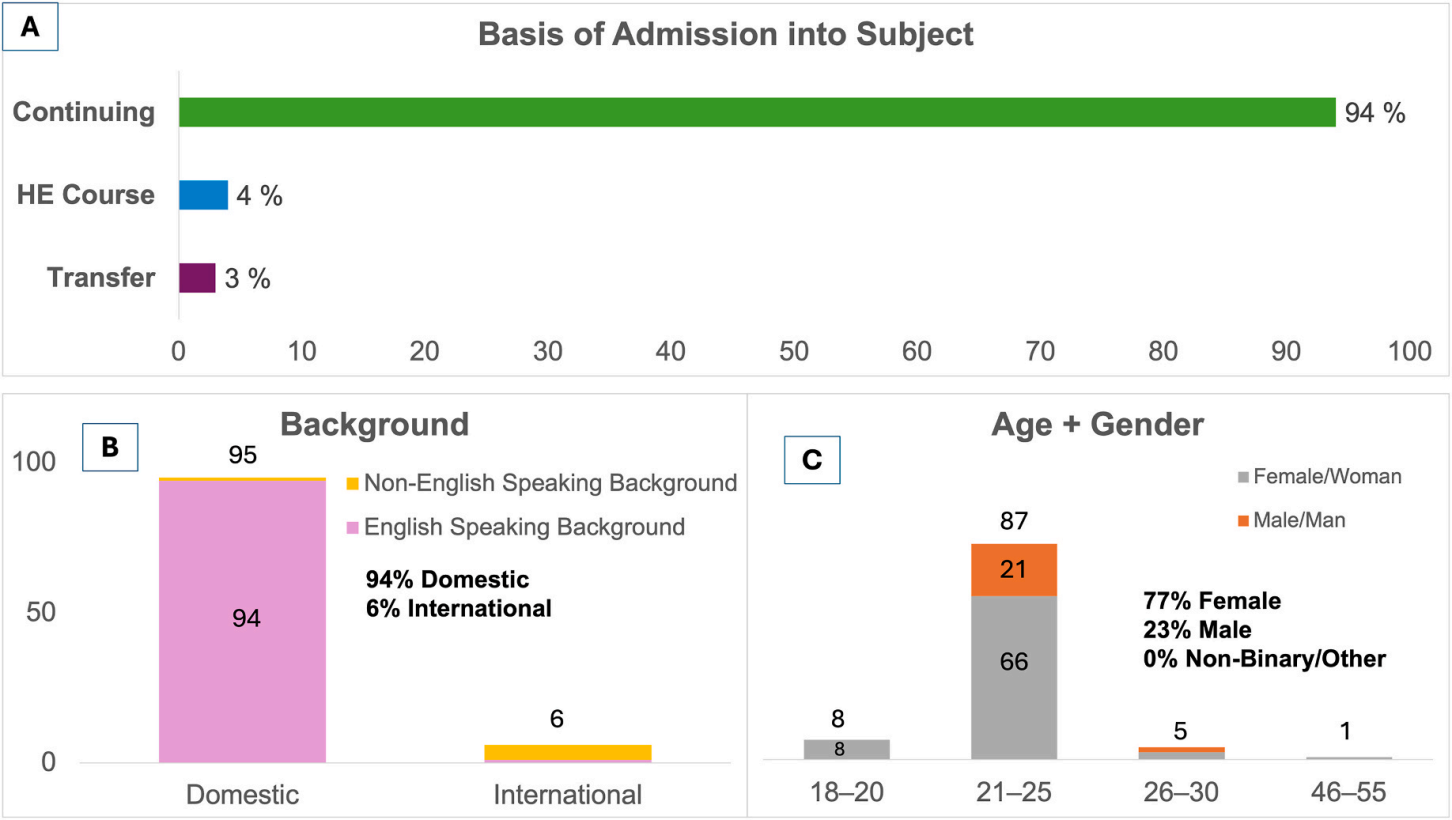
– Major Scene Investigation cohort details. This image illustrates (A) the basis of admission into the subject, (B) student background, and (C) age and gender of the cohort.

While demographic information was not collected as part of this research, the student cohort was composed of 101 students (77 % female, 23 % male), 94 % domestic and 6 % international. 93 % of the students were continuing, 4 % were from a different university, and 3 % had transferred from a different programme within the university (refer to Fig. 2).

### 2.2 Assessment design & context

The subject in which this study was implemented was *Major Scene Investigation*, a second-year core subject for students in the Crime Scene Major in the Bachelor Forensic Science at the University of Technology Sydney. This subject can also be taken as an elective for students in different forensic science majors. In this study, we piloted integrating a new assessment so that students could develop their digital literacy and critical thinking skills. It involved the submission of a portfolio of evidence in Portfolium – a university approved platform that allows students to create a portfolio in various formats.

Students submitted five reflections during the span of the 12-week subject and were encouraged to submit each reflection soon after the practical case-scenario sessions, to ensure that their experiences of using Copilot were fresh in their minds. Students were also encouraged to adhere to a cycle of reflection so that their thoughts could be well structured and organised. To foster critical thinking, students were asked to draw on reliable academic literature such as peer-reviewed journal articles or government reports to better understand both the advantages and disadvantages of embedding GenAI into forensic science workflows, but also to familiarise themselves with current discussions around its ethical and responsible integration in operational settings.

Students had eight 3-h practical sessions where they had to manage and process a simulated crime scene in a team of four to five people. Students were introduced to online Canvas materials that introduced Copilot as a tool from week 1 of term. In the first practical session, students were not asked to use Copilot but were asked to consider how this tool may prove valuable if it was to be used. From week two, students were asked to utilise Copilot during these sessions to gauge whether it could be utilised as a decision-support tool in crime scene contexts and/or to expedite certain crime scene-based processes. Copilot was chosen as a platform because it was a university-approved system and accessible to all students.

This process was designed so that students could engage critically with GenAI's outputs. Students were strongly encouraged to cross-check Copilot's outputs against reliable sources, never taking outputs at face value. Students were also asked not to share any case-sensitive information given during the simulated police briefing and throughout the crime scene examination process with Copilot.

#### 2.2.1 Pedagogical framework

To address the challenges previously identified across both higher education and forensic science contexts, this study is situated within Kift's transition pedagogy principles alongside Universal Design for Learning (UDL) [[33], [34], [35]]. These frameworks collectively informed the design of an assessment approach that supports students' progression from foundational knowledge to applied, practice-based competencies, while ensuring accessibility, inclusivity, and meaningful engagement.

In line with the Assessment Principle of transition pedagogy, the assessment incorporated a structured portfolio approach in which students submitted iterative reflections across the semester. This approach positioned assessment as an ongoing process threaded through the semester instead of a single outcome, enabling formative feedback and encouraging students to engage critically with both AI outputs, feedback, and their own reasoning.

The design also reflects key UDL principles by providing multiple means of engagement, representation, and expression. Students were given flexibility in how they used GenAI and how they presented their portfolio of evidence (e.g. video, audio, or written formats), supporting diverse learning preferences and promoting inclusive participation. The use of institutionally approved tools ensured equitable access, while collaborative crime scene simulations created opportunities for active, experiential learning and peer interaction.

The assessment was designed to promote learner agency by encouraging students to make informed decisions about how and when to use GenAI, and to critically interrogate its outputs in relation to forensic science practice. Through structured reflection, application, and feedback, students were supported in developing transferable skills in critical evaluation, ethical reasoning, and professional judgement, preparing them to engage with GenAI responsibly across academic and real-world forensic contexts.

### 2.3 Data analysis

Reflections were analysed by thematic analysis. Clarke and Braun's six-phased approach to thematic analysis was followed [36], whereby reflections were initially coded, and key themes that emerged from the data were identified. Two different members of the research team underwent the process of coding and theme identification independently to enhance the validity and rigour of this study and minimise subjectivity. The two researchers convened once they had finalised this process to identify commonalities and/or divergence in the theme identification process. While one researcher examined all student reflections, the other focused on a subsample. Four key themes were identified through a mixture of an inductive and deductive approach, which was guided by previous literature, but was flexible to identify new emerging themes not previously considered.

## 3 Results

The following sections have been organised according to the four core themes that emerged through the reflections’ analysis process.

### 3.1 Negotiating trust in GenAI: engagement, ambivalence, and resistance

This theme examines how participants initially oriented themselves to the introduction of GenAI within complex, time-pressured forensic decision-making contexts. Participants' responses reflected varying patterns of engagement, ambivalence, and resistance. Many participants initially approached GenAI with curiosity and enthusiasm, although this was often tempered by scepticism. Early excitement often diminished once the tool was introduced into practical crime scene sessions, where GenAI was perceived as an additional cognitive demand within an already complex investigative environment that students perceived to have well-established decision-making routines. This was frequently described as the tool being “one more thing” to manage, alongside multiple competing tasks, as noted in two participants’ perspectives below.

**Participant 6:** "*I don’t believe Copilot can help speed up my work at a crime scene. Most scenes have a time frame an examination must be completed in and stopping to ask Copilot how I should complete ‘X, Y, Z’ takes away valuable time.*"

**Participant 14:** "*I know that it can reduce the amount of admin work I need to do, but there are inconsistencies within that that require manual checking. This adds rather than takes away time on the job*."

These feelings reflected a process of destabilisation, where existing cognitive workflows were temporarily unsettled as students attempted to integrate unfamiliar technologies into practice. This characterisation challenges dominant narratives of AI as a frictionless educational support tool that can seamlessly be integrated to enhance student performance and knowledge retention [37], instead positioning the use of GenAI as a potential source of cognitive and attentional disruption, particularly when used in practical, high-pressure contexts. However, it is also worth noting that participants may have experienced similar attitudes towards any novel and cognitively demanding assessment, even when unrelated to GenAI.

Nevertheless, from a decision-making perspective, these findings suggest that the introduction of GenAI may initially intensify – rather than reduce – cognitive load under conditions of time pressure and complexity. Cognitive load theory highlights the limited capacity of working memory to process and integrate multiple information streams [37]. This means that the addition of AI-generated outputs may impose further peripheral demands on already constrained attentional and processing resources. In safety- and time-critical environments, such increases in cognitive load have been shown to negatively impact decision-making performance and efficiency, particularly when uncertainty must be actively interpreted and managed [38]. This effect is likely to be more pronounced among students or novice crime scene examiners, who are still developing the domain-specific schemas required to efficiently filter and prioritise information, but could affect any crime scene practitioner, given that crime scene processing is known to require sustained and increased cognitive load, under uncertain, dynamic conditions and under pressure [39].

It is noteworthy, however, that for some participants, this initial orientation shifted following an in-person workshop that clarified assessment expectations and contextualised the intended role of AI technologies within forensic decision-making and in forensic science more broadly. This structured and scaffolded guidance appeared to support a recalibration of engagement and motivation, with participants demonstrating greater willingness to experiment with GenAI in a cautious but constructive manner. Previous studies have shown that student engagement can be captured when educators demonstrate care and genuine investment in students’ learning (e.g. by providing scaffolded, structured, experiential learning opportunities), creating a reciprocal engagement cycle between staff and students [40]. In turn, this increased motivation can translate into improved academic performance and deep learning acquisition [41], which appeared to be reflected in this study. While scepticism remained, these individuals began to explore context-specific and, at times, creative aspects of the tool, extending beyond educator-provided examples. This shift suggests that early uncertainty is not fixed but can be mitigated through clearer framing of GenAI purpose and use, enabling more calibrated and critically informed engagement. These findings also indicate that, for the participants, GenAI trust is actively shaped through context, guidance, and experience.

In contrast, a smaller group of participants expressed more persistent resistance towards Copilot, even after the workshop. These students acknowledged the potential value of AI within forensic science more broadly but described GenAI as impractical and disruptive to workflow efficiency, particularly under time constraints. This group of students reported discontinuing its use during practical sessions despite this being required for assessment. However, rather than representing simple reluctance or rejection, this resistance was frequently framed as concerns that reliance on GenAI could displace meaningful cognition, learning, and human instruction, with two participants stating:

**Participant 17:** "*Relying too heavily on automation could make me less attentive to detail or procedural accuracy*".

**(Participant 11):***"I’m a big believer that the prevalence of AI in the current world is limiting our ability to think for ourselves".*

Although these views are similarly captured in academic literature [3], it seemed to go against the prevalence of use reported elsewhere [28,29], further illustrating the complexity, selectivity, and individuality of student engagement.

### 3.2 GenAI as a contingent cognitive partner – boundary setting in decision-support

This theme examines how participants defined and operationalised the role of GenAI as a decision-support tool within crime scene reasoning processes, rather than as an authority for final conclusions or decisions. The majority of participants’ accounts illustrate how GenAI was selectively integrated to assist mostly with preparatory, organisational, and sense-making tasks, and most participants felt that ultimate interpretative and decision-making responsibility ought to be retained by human examiners.

Many participants reported that GenAI supported aspects of forensic reasoning by enabling brainstorming (e.g. identification of potential hazards in a scene, appropriate identification of crime scene search strategies given specific room configurations, trace collection and packaging strategies), clarifying terminology and, when possible, learning about jurisdiction-specific protocols, as well as structuring their approach and workflow to complex tasks (e.g. establishing potential forensic links across simulated cases through adoption of a forensic intelligence approach). One participant reported:

**Participant 1:** "*Across the practical sessions throughout the course of M[ajor] S[cene] I[nvestigation], Copilot demonstrated clear strengths in structured, factual tasks, such as hazard management and contamination protocols, providing a faster and in-depth analysis.*"

GenAI tools used for similar academic tasks have been widely reported in literature [3,[42], [43], [44]] but, more importantly, are being utilised for similar purposes in professional policing contexts [6]. This suggests that, for students, developing practical experience with this type of tool will already be an advantage once they transition into the workforce.

Participants described using Copilot to organise thoughts, identify relevant considerations that they would have otherwise missed, and scaffold their reasoning and further learning during crime scene activities and beyond these structured practical sessions. For instance, when testing out Copilot as a tool that could establish links between connected cases, a participant stated:

**Participant 2:***“Copilot's contribution was conceptually accurate, drawing attention to the connection between chemical evidence and criminal exploitation"*.

Several participants highlighted that, over time, they realised the importance of prompt engineering, which is defined as the steering mechanism that helps in shaping the usefulness of AI responses [45]. Participants observed that refining prompts allowed them to obtain more targeted and relevant outputs.

**Participant 18:** "*Initially, I felt disappointed when the first response was irrelevant, as it reflected a communication gap between forensic terminology and GenAI’s interpretation. However, this encouraged me to reconsider how I ask my questions with specificity and disciplinary context when using GenAI in forensic science. After refining the questions step by step, I felt satisfied and more confident using Copilot to produce a coherent, forensically accurate answer consistent with professional practice.*"

Although initial integration of GenAI during practical sessions was often perceived as impractical or distracting, some participants reported that this interaction became more intuitive over time as they developed familiarity with both the tool, as well as its advantages and limitations. What was noticed when evaluating Portfolio entries was that prompts often lacked context, structure, and subsequent improvement. In turn, ensuing reflections and critical thinking of outputs was also often limited, despite being necessary steps in fruitful human-AI collaborations and knowledge co-construction [46]. It is worth noting that the lack of appropriate prompt optimisation occurred despite providing students with examples and resources to develop these skills.

Furthermore, for participants, GenAI was rarely positioned as a source of authoritative answers. Instead, participants consistently described its use as supporting and occasionally enhancing their own reasoning processes, rather than replacing them. Participants emphasised the need to independently evaluate AI-generated information, cross-check outputs, and apply disciplinary knowledge when interpreting results – which is well aligned with recommendations to ensure critical AI literacy is developed [47]. This selective and bounded use suggests an organic tendency or perhaps emerging awareness towards distinctive decision-support versus decision-making, with GenAI occupying a subsidiary role within the broader investigative workflow. This perspective is observed in other literature that very much positions AI tools as providing support, rather than autonomous, final, decision-making [16,17,48].

### 3.3 Ethics, bias, and accountability in GenAI use

This theme examines how participants reasoned about ethical boundaries and professional accountability when engaging with GenAI in crime scene decision-making contexts. Instead of focusing solely on the functionality of GenAI, participants’ reflections highlighted concerns about accuracy and legitimacy of outputs, responsibility, and defensibility of decisions informed by AI-generated information.

Most participants expressed uncertainty regarding the ethical appropriateness of using GenAI in relation to both assessment and crime scene-related tasks. This was despite receiving structured guidance on its intended use. This perhaps reflected deep tensions surrounding the integration of AI into domains governed by professional standards and evidential scrutiny. While most participants avoided sharing case-sensitive information with Copilot due to concerns around confidentiality and data security, some engaged in exploratory use by inputting simulated case materials to understand the tool's capabilities, despite being instructed not to do this. These interactions revealed important limitations from a student perspective – while prompt refinement could generally generate appropriate suggestions, outputs were often generic, lacked jurisdictional specificity, or contained inaccuracies and hallucinated details. However, upon closer educator-based inspection, many prompts were not specific enough or lacked vital context relevant to the anticipated output. Students often phrased their prompts to Copilot in the same way they would ask teaching staff questions inside the classroom. They assumed GenAI could access and understand the contextual complexities of their scenario, without providing the necessary information to give an informed response. Nevertheless, such experiences contributed to growing student scepticism regarding the reliability and appropriateness of GenAI in forensic contexts.

Participants consistently expressed discomfort with relying on GenAI for substantive forensic judgements, even when acknowledging its potential utility for preparatory or organisational tasks. This reluctance was often framed in a professional accountability context, with participants emphasising the need to be able to justify decisions and withstand scrutiny in forensic science and the broader justice system. The opaque or black box nature of many AI systems was perceived as incompatible with such requirements, limiting their acceptance when applied to high-stakes decision-making environments. As a result, participants drew a clear boundary between low-stakes support tasks and high-stakes interpretative decisions, reinforcing the principle that accountability cannot be delegated to AI systems.

Concerns about ‘cheating’ were frequently articulated, but these concerns went beyond academic integrity to encompass questions of responsibility and ownership of decisions; participants questioned who should be held accountable if AI-generated information influenced an incorrect or unjust outcome, emphasising that accountability should remain with the human decision-maker. Notably, a very small number of participants expressed strong disdain for universities' stance to introduce GenAI tools to university learning and assessments. This reflected a clear misalignment around the role of technology in their learning journey and their professional identity. It is worth noting that when participants experienced such strong aversion towards AI, grounded on feelings of ethics and morality, it prevented participants from learning about the opportunities and limitations of such systems. However, this resistance was not always fixed and, in some cases, clearer articulation of the relevance of AI literacy to future professional practice prompted more open engagement. Participant reflections demonstrated attempts to establish defensible and professionally acceptable boundaries of GenAI use. This aligns well with concerns in forensic governance literature regarding transparency, auditability, and the attribution of responsibility in AI-assisted decision-making [14,16,17,19]. Participants' ethical reasoning was closely intertwined with their practical engagement with the tool, shaping patterns of selective reliance, cautious use and, in some cases, complete avoidance.

### 3.4 From experimentation to critical vigilance: developing AI literacy

This theme examines how participants believed they have developed AI literacy over time, moving from initial experimentation and uncertainty towards more critical, selective, and context-sensitive engagement with GenAI.

For clarity, and as defined in Ref. [49], AI literacy is ‘the ability to critically engage with AI technologies, understand their societal and ethical implications and apply them responsibly. It integrates knowledge, skills, and attitudes across technical applicational, critical thinking, ethical, social, integrational, and legal dimensions, with variations in emphasis depending on the disciplinary context’. The authors in [49] identify four competency levels (unaware, beginner, intermediate, expert) that could be applied to the present study, whereby most students went from an unaware or beginner level to an intermediate one. In a similar vein [50], indicate that students who typically don't perform as well will require more scaffolded and foundational instruction, those with intermediate level of proficiency would benefit from problem-solving-based learning, and those with more advanced competency require learning based on critical assessment. For the present cohort, this competency process did not represent linear engagement and acquisition of technical skills and, instead, for most students, critical AI literacy emerged gradually over time, and encompassed not only functional or technological competence, but the ability to evaluate, interrogate, and appropriately apply AI-generated outputs within forensic science contexts.

The 12-week journey to teach students about the ethical and responsible use of AI seemed fit for purpose, particularly for those students who were open and flexible to learning about the tool and how it can be utilised in practice to potentially support diverse forensic workflows. Students were able to identify the types of tasks for which it would be acceptable to utilise Copilot both in academia and crime scene investigations and developed a practical understanding of prompt engineering to improve GenAI outputs. They also experienced the evolution of this platform in just a few months – tasks it couldn't perform at the start of the semester, it could complete by the end of it. Participants reported that while initially learning about how it could be incorporated in practical sessions to support their learning and expedite or support scene decision-making processes felt counter-productive, impractical, and tedious, towards the end of the 12 weeks it became second nature.

AI has been identified as a potential cognitive amplifier or inhibitor [51]. As referred to in sections 3.1, 3.2, students that persevered with the Copilot system both to brainstorm and to explore it as a tool for decision support, were able to engage with it more deeply. This is likely attributed to the tool being integrated in such a way that fostered autonomy, problem-solving and critical thinking [51]. Other studies in the literature suggest that AI-driven technology can attenuate the cognitive load of students in various ways; by scaffolding complex concepts, adjusting instructional materials, and through provision of instant feedback [37]. This reduction in cognitive overload has been previously associated with improvements in learning outcomes.

Nevertheless, it's worth noting that while this was a university assessment task, there were some students that altogether refused to utilise Copilot during practical sessions because they had already formed an opinion of its value – or lack thereof – prior to the start of the subject. These students were alarmed to learn that GenAI formed part of the assessment journey and that it may be utilised to support real-world forensic workflows. These students therefore failed to acquire the AI literacy that educators hoped students would develop and this was reflected in their final reflections where, for instance, it was stated that they did not wish to form part of the training dataset for Copilot, without realising that the enterprise version of Copilot does not rely on inputs to train their models. Yet they stated using ChatGPT to improve their work, which does, unless specifically selected, rely on data inputs to train models. The failure to acquire critical AI literacy was also apparent in the lack of creativity applied to GenAI integration into the investigative process. Often students would default to the exemplars provided in the first few weeks of classes, not attempting to experiment with new ways the tool could be applied. The lack of engagement with the assessment tasks and feedback encountered in some students in this study was not entirely surprising or uncommon (see for instance Ref. [52]), as it can be intricate to capture every students' attention and foster motivation with a multilayered and complex assessment task.

## 4 Discussion

The present findings suggest early calibration and boundary-setting when establishing a human-AI collaboration in crime scene contexts and higher education assessments. Participants used GenAI to reduce cognitive burden in the initial stages of crime scene-based processes that required decision-making, and reserved high-stakes judgement and accountability-laden decisions for themselves or to be compared against the judgement of an instructor for sense-checking. This approach very much aligns with forensic science literature that emphasises AI should be used as an augmentative tool rather than an autonomous decision-maker, reinforcing the central role of human expertise, contextual understanding, and professional judgement in evidentiary processes [16,17].

The findings in this study differ from existing accounts of GenAI adoption in higher education environments, which often associate positive attitudes with increased engagement and use. Participant reflections suggest that acceptance of these systems does not necessarily translate into meaningful sustained engagement, particularly within high-stakes, cognitively demanding environments such as crime scene investigation. This may reflect the disciplinary context of forensic science, where students are routinely encouraged to critically evaluate information, justify decisions, and carefully consider the consequences of error, bias, and uncertainty. While prior research indicates that positive attitudes towards GenAI may increase the likelihood of its use, they do not necessarily support the development of deeper AI literacy and may, in some cases, serve to neutralise or justify its misuse [53]. Conversely, greater familiarity with AI concepts has been associated with more critical and informed engagement [54]. More generally, resistance to educational technologies is thought to be associated with underlying anxiety, stress, and uncertainty [55,56], as well as wider tensions associated with exposure to digital change in learning environments [57]. Deacon et al. (2025) [56] further identify key drivers of resistance to technology, including feeling overwhelmed, fear of technology and of being replaced, and ideological feelings surrounding learning practices, all of which are reflected in the present findings. Within this study, such responses appear closely tied not only to emotional reactions towards AI in general but to the cognitive and epistemic demands of operating within complex and high-pressure forensic decision-making environments.

The findings suggest that introduction to GenAI should be presented in scaffolded and structured ways if it is to be used effectively in high-pressure and time-sensitive investigative environments. Viewed through the perspective of Kift's transition pedagogy [58], these findings emphasise the importance of structured scaffolding during early-stage learning, where students are simultaneously adapting to disciplinary expectations and developing foundational competencies. Instead of fostering immediate efficiency in decision-making contexts, GenAI may initially destabilise learning processes, reinforcing the need for guided, scaffolded engagement - preferably from year one as suggested in the First and Further Year Experience Program - that supports both cognitive load management and the development of critical, independent judgement.

The introduction of GenAI into already demanding environments may heighten perceived workload and uncertainty, shape perceptions of usefulness and influence willingness to engage. Factors such as interface usability, performance, clarity of purpose, and consistency of institutional guidance further mediate these early experiences, affecting attention, satisfaction, and perceived value during task performance [59,60]. These findings confront implicit assumptions that the introduction of GenAI into academic or professional contexts is experienced as either immediately beneficial or readily adoptable, instead revealing a more complex process of negotiation and adjustment, in which adoption unfolds through ongoing tensions between the tool's perceived value and constraints, uncertainty, trust, and ethical and contextual concerns associated with using it within academic and crime scene contexts [44,61,62].

It is also worth noting that this initial uncertainty and resistance may be understood in light of earlier institutional responses to AI – such as widespread bans on its use in assessment following the release of GPT-3.5 – which positioned AI primarily as a threat to academic integrity rather than a tool for learning [43]. The subsequent shift towards encouraging responsible and ethical AI use [30] may have contributed to participants’ ambivalence, as students navigated competing narratives of prohibition and adoption. This tension may help explain the confusion and caution observed in this study, particularly among those transitioning into higher education environments where expectations around AI use are still evolving.

Overall, this study has shed further light on the potential and inherent limitations of AI in forensic science as experienced and perceived by higher education students. While AI can enhance efficiency, consistency, and scalability, it does not currently resolve challenges relating to bias, interpretation, accountability, and evidentiary defensibility. Instead, AI may influence how forensic decisions are made, placing increased responsibility on practitioners to critically evaluate, contextualise, and justify AI-assisted outputs. This highlights the need for forensic science education to move beyond technical awareness, towards preparation of future practitioners who can exercise informed, ethical, and defensible judgement when engaging with AI-enabled decision-support tools in professional practice. This, in turn, provides a clear rationale for assessment approaches that embed GenAI within authentic, simulation-based learning environments, enabling students to engage with these technologies in context while developing informed and justifiable decision-making capabilities.

### 4.1 Limitations

This study has several limitations that ought to be considered when interpreting the findings.

A potential selection bias should be acknowledged, given that participation in the research component was voluntary, and not all students enrolled in the subject consented to the use of their reflections for research purposes. As a result, the findings do not reflect the perspectives and experiences of all students who were asked to engage with GenAI in assessments or forensic science contexts. Consequently, the findings should not be interpreted as representative of all forensic science students, but as indicative of how individuals may engage with AI-assisted decision-support tools during early stages of professional development. It is also worth noting that this study occurred within institutional contexts that were characterised by emerging and evolving university guidance which, to students, also appeared to be inconsistent with prior guidance regarding acceptable GenAI use. This may have further impacted their experience and engagement with these tools.

In addition, the data analysed in this study was derived from reflective portfolio submissions that formed part of an assessed task. Although reflections were analysed only after subject completion, it remains possible that students moderated their responses due to their awareness of assessment or perceived expectations. To mitigate this, reflections were anonymised prior to analysis and independently coded by more than one researcher; however, the influence of assessment context cannot be fully eliminated.

Finally, this study focused on simulated crime scene examinations. While simulation-based learning provides a controlled environment for evaluating decision-making under uncertainty, it does not replicate the full operational, legal, and emotional pressures of real forensic casework. Accordingly, findings should be interpreted as reflecting how individuals at an early stage of forensic training interact with AI-enabled support in an unrestricted format that allowed for experimentation, rather than as direct analogues for practicing crime scene examiners or forensic science professionals.

Despite these limitations, the study provides valuable insight into human-AI interaction, trust, and judgement formation in forensic decision-making contexts, informing ongoing discussions around the responsible integration of GenAI into forensic science workflows and education.

## 5 Conclusions and future outlook

This study embedded a university-approved GenAI tool – Copilot – into a second-year forensic science subject to explore how early-stage decision-makers evaluated AI-generated information, negotiated ethical boundaries, and calibrated trust and responsibility within high-fidelity crime scene simulations.

Generally, the findings indicate that participants did not engage with GenAI in an uncritical manner and did not use it as a substitute for their own cognition or professional judgement. Instead, participants demonstrated a cautious and selective approach, positioning GenAI as a conditional decision-support tool rather than as an authority for crime scene-based decisions or conclusions. Although initial reactions were often characterised by uncertainty, frustration, or scepticism, many students developed more nuanced understandings of the tool's capabilities and limitations over time. Most students reported overcoming initial frustrations within the 12-week duration of the subject, and reported Copilot integration into crime scene workflows becoming second nature. This was particularly evident in their differentiation between low-stakes and high-stakes decision contexts, with reliance on GenAI noticeably affected when accountability and evidentiary consequences were perceived to be greater.

Ethical reasoning and professional accountability emerged as central to how students evaluated the appropriateness of using GenAI. Participants noted concerns around explainability, justification of decisions, potential for hallucinations, and attribution of responsibility, which closely mirrored those raised within the forensic AI governance literature [6,14,19,30]. This suggests strong alignment between student reasoning and professional values. From an educational perspective, the findings demonstrate the value of embedding GenAI within authentic, contextualised assessments that foreground problem solving, critical and technological thinking rather than AI-driven efficiency gains alone. The use of high-fidelity crime scene simulations, paired with iterative reflective portfolios, provided students with opportunities to experience and interrogate the cognitive and ethical tensions associated with GenAI-supported decision-making. This approach reinforced the development of critical AI literacy, enabling students to articulate when GenAI may be useful, when it should be approached with caution, and when it may be inappropriate to rely upon it altogether.

Future research should explore whether similar patterns of engagement and boundary-setting are observed among practicing forensic professionals, and how organisational culture, operational pressures, and governance frameworks shape AI-assisted decision-support in casework. Longitudinal studies tracking the development of AI literacy across forensic science training and into professional practice would also provide valuable insight into how judgement around AI has evolved over time. It is undeniable that – whether we like it or not – GenAI will continue to reshape educational, investigative, and professional environments, and that students, practitioners, and their governing institutions will increasingly be required to adapt, learn, and engage with AI technologies. In this evolving landscape, the way forward is not to avoid AI, but to prepare users to critically evaluate, appropriately constrain, and responsibly integrate these tools within forensic processes in a manner that maintains accountability, transparency, and defensible decision-making.

## CRediT authorship contribution statement

**Paula Tarttelin Hernández:** Conceptualization, Data curation, Formal analysis, Funding acquisition, Investigation, Methodology, Project administration, Resources, Writing – original draft, Writing – review & editing. **Alicia Haines:** Conceptualization, Funding acquisition, Project administration, Writing – review & editing. **Vincent Mousseau:** Conceptualization, Funding acquisition, Methodology, Writing – review & editing. **Madysen Elbourne:** Data curation, Formal analysis, Investigation, Writing – review & editing. **Teneil Hanna:** Writing – review & editing.

## Declaration of competing interest

The authors declare the following financial interests/personal relationships which may be considered as potential competing interests:Authors declare that they have no known competing financial interests or personal relationships that could have appeared to influence the work reported in this paper.
