# Supplementary material for: Generative artificial intelligence in forensic science education: Student perspectives on AI-supported crime scene decision-making
Source: Forensic Sci Int Synerg. 2026 Aug 6;13:100723. doi: 10.1016/j.fsisyn.2026.100723 (PMC13475356; doi:10.1016/j.fsisyn.2026.100723)
Supplement: Supplementary file 1 [file mmc1.docx]

Table S1: Outline of key milestones of GenAI

| **Date** | **Category** | **Milestone** | **Further information and references** |
| --- | --- | --- | --- |
| Nov 2022 | Global AI | ChatGPT released by [OpenAI](https://openai.com/index/chatgpt/) |  |
| Dec 2022 | Global AI | ChatGPT reaches 1 million users within 5 days | K. Curran, E. Curran, J. Killen and C. Duffy, The role of generative AI in cyber security, Metaverse (2024) Vol. 5 Pages 2796 DOI: 10.54517/m2796 |
| Jan 2023 | Higher Education | Universities rapidly adapt assessment and academic integrity policies in response to ChatGPT | Cotton, D. R. E., Cotton, P. A., & Shipway, J. R. (2023). Chatting and cheating: Ensuring academic integrity in the era of ChatGPT. Innovations in Education and Teaching International, 61(2), 228–239. https://doi.org/10.1080/14703297.2023.2190148  Susnjak, T. (2022). ChatGPT: The End of Online Exam Integrity? arXiv:2212.09292. <https://doi.org/10.48550/arXiv.2212.09292>  <https://www.unesco.org/en/articles/guidance-generative-ai-education-and-research> |
| Feb 2023 | Global AI | [Microsoft Copilot](https://www.microsoft.com/en-au/microsoft-copilot?utm_source=chatgpt.com) released | Expansion of GenAI into productivity tools |
| Mar 2023 | Global AI | GPT-4 [released](https://arxiv.org/abs/2303.08774) | Major increase in AI capability |
| Apr 2023 | Assessment Integrity | [Turnitin](https://www.turnitin.com/press/turnitin-turns-on-ai-writing-detection-capabilities-for-educators-and-institutions-southeast-asia) introduces AI-writing detection | First widely adopted AI-detection tool |
| Jul 2023 | Australian Sector | Australian Universities Accord [Interim Report](https://www.education.gov.au/australian-universities-accord/resources/accord-interim-report) 2023 identifies AI as a major challenge/opportunity | National policy context |
| Jul 2023 | UTS | [Generative AI Study Guide Canvas module launched](https://www.lib.uts.edu.au/whats-on/news/your-go-to-guide-for-helping-students-understand-ethical-use-of-genai) | Early student guidance initiative |
| Nov 2023 | Global AI | GPT-4 Turbo and Custom GPTs released | Shift toward personalised AI tools |
| Feb 2024 | UTS | [Free secured GPT-4 access provided through Copilot](https://educationexpress.uts.edu.au/blog/2024/02/07/working-securely-with-microsoft-bing-chat-copilot/) | Institutional adoption strategy |
| Feb 2024 | UTS | [GenerativeAI@UTS blog](https://educationexpress.uts.edu.au/blog/2023/02/07/generative-ai-uts-engagement/) launched | Staff and student guidance |
| May 2024 | Global AI | [GPT-4o](https://openai.com/index/hello-gpt-4o/) released | First mainstream multimodal AI |
| Jun 2024 | Regulation | [TEQSA Request for Information (RFI) on GenAI](https://www.teqsa.gov.au/about-us/news-and-events/latest-news/information-request-issued-ai-risk-mitigation) | National regulatory response |
| Jul 2024 | UTS | [UTS response to TEQSA RFI submitted](https://wordpress-futures-prod.s3.ap-southeast-2.amazonaws.com/wp-content/uploads/2024/08/29145413/UTS-TEQSA-RFI-Response-July-2024-1.pdf) | Institutional governance milestone |
| Jul 2024 | UTS | Assessment reform and whole-of-course transformation commences | Major curriculum response  Internal UTS communication |
| Sep 2024 | Australian Sector | [Two-Lane Assessment Approach introduced](https://educational-innovation.sydney.edu.au/teaching@sydney/program-level-assessment-two-lane/) at University of Sydney | Influential sector model |
| Nov 2024 | UTS | [Five Principles for Effective and Ethical Use of Generative AI](https://educationexpress.uts.edu.au/collections/artificial-intelligence-in-learning-and-teaching/resources/five-principles-for-effective-ethical-use-generative-ai/) released |  |
| Nov 2024 | UTS | Mosaic Approach to Assessment Integrity introduced | Assessment redesign framework  Internal UTS SharePoint site |
| 2025 | UTS | Secure ID-verified assessments implemented | Transition to secure assessment ecosystem  Internal UTS SharePoint site |
| 2025 | UTS | Assessment transformation embedded across courses | Internal UTS SharePoint site |
| Aug 2025 | Global AI | [ChatGPT 5.0](https://openai.com/index/introducing-gpt-5/) released | Continued evolution of AI capability |
